# Neutrophil-to-Lymphocyte Ratio as a Predictor of Disease Severity and Mortality in Coronavirus Disease 2019: Prospective Study From Central India

**DOI:** 10.7759/cureus.23696

**Published:** 2022-03-31

**Authors:** Bharatsing D Rathod, Dnyanesh Amle, Rajashree S Khot, Kiran Kumar Prathipati, Prashant P Joshi

**Affiliations:** 1 General Medicine, All India Institute of Medical Sciences, Nagpur, IND; 2 Biochemistry, All India Institute of Medical Sciences, Nagpur, IND; 3 Otorhinolaryngology, All India Institute of Medical Sciences, Nagpur, IND

**Keywords:** neutrophil-to-lymphocyte ratio (nlr), cytokine storm, mortality, severity, covid-19

## Abstract

Background: Clinical presentation of coronavirus disease 2019 (COVID-19) varies from an asymptomatic state to severe disease characterized by acute respiratory distress syndrome, respiratory failure, thrombosis, and multi-organ dysfunction syndrome. The neutrophil-to-lymphocyte ratio (NLR) has been reviewed as one of the laboratory factors that have been proposed to predict the severity of disease and mortality in COVID-19 pandemic.

Aim and objectives: To evaluate the association between NLR and the disease severity and mortality in COVID-19.

Materials and methods: After approval from Institutional Ethics Committee, this prospective cohort study was carried out in a tertiary-care teaching medical institute of Central India. COVID-19 patients of the age group 18 years and above admitted during the study period were included. Cases were categorized into four groups as asymptomatic (Group A), mild (Group B), moderate (Group C), and severe (Group D) based on clinical symptoms, respiratory rate, oxygen saturation, and chest imaging. NLR was calculated by doing a complete blood count at the time of hospitalization by the Mindray BC-6000 auto hematology analyzer. The outcome of the disease was classified as recovery and death during hospitalization. Receiver operating characteristic (ROC) curve analysis was used to assess the ability of NLR at admission to predict severe COVID-19 or mortality. Ordinal regression analysis was used to assess the impact of NLR on disease severity and mortality.

Results: Mean NLR was significantly higher in the severe COVID-19 group as compared to the mild/moderate group and in deceased as compared to discharged cases. ROC curve analysis revealed NLR to be an excellent predictor of disease severity as well as a prognostic parameter for risk of death. NLR was found to be a significant independent positive predictor for contracting the severe disease (Odd’s ratio 1.396, 95% CI=1.112-1.753, p=0.004) and mortality (Odd’s ratio 1.276, 95% CI=1.085-1.499, p=0.003).

Conclusion: High NLR was significantly associated with the disease severity and mortality in COVID-19.

## Introduction

The recent coronavirus disease 2019 (COVID 19) pandemic has resulted in significant morbidity and mortality worldwide. It has affected more than 452,201,500 individuals and caused more than 6,029,850 deaths all over the world to date [1]. This number is expected to rise further due to the possibility of subsequent waves. Presentation of COVID-19 varies widely among individuals, remaining as a mild illness affecting the upper respiratory tract only in the majority of cases. But in a few cases, it can progress to involve the lower respiratory tract, worsening to acute respiratory distress syndrome (ARDS), respiratory failure, multiple organ dysfunction syndrome (MODS), and subsequent mortality. Many risk factors have been associated with the severity of disease and mortality in COVID-19 [2].

Due to the overburdening of the healthcare system in a pandemic situation, it is important to do triaging of the COVID-19 cases, so that individuals having risk factors for progression to the severe disease can be given proper medical care on a priority basis. Thus, it is important to find predictors for severity and mortality in COVID-19. The host immune system plays a very important role in the pathogenesis of COVID-19. Aggressive inflammatory response to severe acute respiratory syndrome coronavirus 2 (SARS-COV-2) can lead to a “cytokine storm” correlating directly with lung injury, multi-organ failure, and unfavorable prognosis of severe COVID-19 [3,4]. Emerging evidence suggested that peripheral blood neutrophil-to-lymphocyte ratio (NLR) can be used as a marker of systemic inflammation [5,6]. NLR has shown good predictive values on progression and clinical outcomes in various diseases such as chronic obstructive pulmonary disease (COPD), cardiovascular disease, and pancreatitis [7-9]. Recently, many studies have reported the role of NLR in differentiating mild/moderate cases from severe COVID-19 cases. Several studies have proposed that NLR can be a reliable predictor of COVID-19 progression and found that elevated NLR was associated with high mortality in COVID-19 [10-14].

NLR is cost-effective, readily available, and easy to calculate laboratory marker. There are very few studies documenting the role of NLR in COVID-19 from this region. With this background, the current study has been carried out to find out the association of NLR with the severity of disease and treatment outcome (recovery or death) among laboratory-confirmed cases of COVID-19 admitted in a tertiary-care teaching medical institute of Nagpur city, Central India.

## Materials and methods

**Figure 1:**
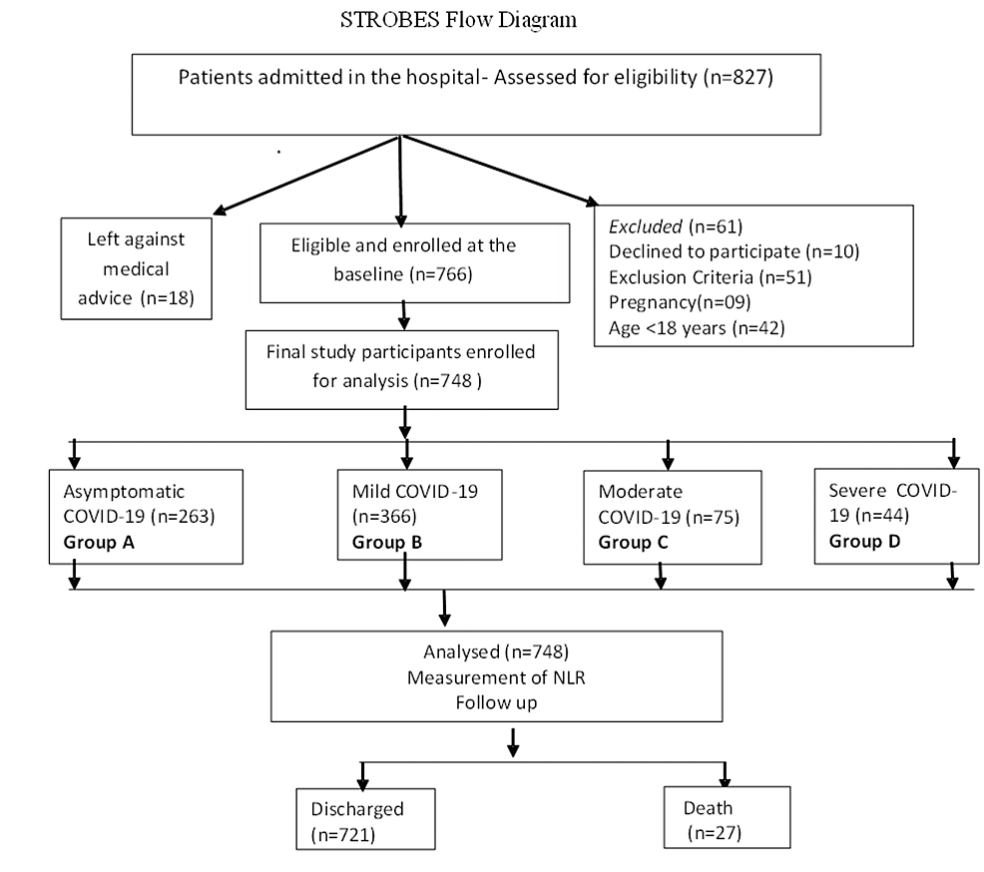
Recruitment and assessment flowchart for the participants NLR: neutrophil-to-lymphocyte ratio; COVID-19: coronavirus disease 2019

This prospective cohort study was carried out in a tertiary-care teaching medical institute of Nagpur city. The study was approved by the Institutional Ethics Committee (All India Institute of Medical Sciences, Nagpur, approval no. IEC/Pharmac/2020/140 dated: July 2, 2020). Nagpur was one of India’s highest disease burden cities, especially during the second wave of COVID-19. All consecutive cases of COVID-19 hospitalized for one year (from July 2020 to June 2021) were included in this study. Patients less than 18 years old, critically ill patients (unable to talk/ give interview), pregnant women, not knowing Hindi/English or Marathi language, and those refusing to give consent were excluded. The participants were recruited within 24 hours of their admission. The data were collected by the investigators using a pre-designed structured proforma. Cases were interviewed about socio-demographic information, details of their current illness, past illnesses including comorbidities, and any substance use patterns. After obtaining medical history, clinical examinations were done and recorded in the schedule. All the interview and clinical examinations were carried out maintaining COVID-19-related government protocol, i.e., social distancing and aseptic procedure. Diagnosis of COVID-19 was made by positive reverse transcriptase-polymerase chain reaction (RT-PCR) test for SARS-COV-2 from nasopharyngeal or oropharyngeal sampling. The reports of those participants who got COVID tested from any government-registered laboratory outside the hospital were also considered valid. Blood samples were collected immediately after their recruitment using institutional standard operating procedures (SOPs), for testing complete blood count, serum ferritin, and C-reactive protein. All diagnostic tests were carried out using the validated technique in accredited laboratories maintaining SOPs. The patients were categorized into four groups as asymptomatic (Group A), mild (Group B), moderate (Group C), and severe (Group D) based on clinical symptoms, respiratory rate, oxygen saturation, and chest imaging (see definition part). NLR was calculated by dividing neutrophil percentage by lymphocytes. The outcomes of the disease were classified as recovery and death during hospitalization (Figure 1).

Operational definitions

The severity of COVID-19 disease was categorized according to the guidelines of the World Health Organization and as per the interim treatment guidelines issued by the Ministry of Health and Family Welfare, Government of India, based on the patients’ respiratory status and oxygen saturation, the cases were divided into four groups: asymptomatic, mild, moderate, and severe [15].

Asymptomatic: No symptoms. Vital parameters: stable, SpO₂ ≥ 94%, no clinical and /or radiological evidence of lower respiratory tract infection/pneumonia.

Mild: Mild symptoms of upper respiratory tract infection, vital parameters: stable, SpO₂ ≥ 94%, no clinical and/or radiological evidence of lower respiratory tract infection/pneumonia.

Moderate: Any one of these 1) respiratory rate ≥ 24/minute, 2) SpO₂ ≤ 93%, along with symptoms like shortness of breath.

Severe: Any one of these 1) respiratory rate > 30/minute, 2) SpO₂ ≤ 90%, along with symptoms like shortness of breath/evidence of ARDS, respiratory failure requiring assisted ventilation, MODS.

Patients were monitored daily throughout their hospitalization course and outcomes were recorded. The patients who did not take the complete course of treatment and left against medical advice were excluded from the study. Main outcome variables include the severity of the illness and treatment outcome (death/discharge).

Ethical issues

Institutional Ethics Committee approval was obtained before initiating the study (IEC/Pharmac/2020/140 dated 2nd July 2020). After approaching the study participants at their convenient time, they were explained the purpose of the study. They were included in the study after obtaining informed written consent. Anonymity and confidentiality were maintained throughout the process. Separate permission was obtained for blood collection and testing. It was ensured that they could opt-out at any time during study and their routine services will not be hampered if they deny participating. The data were stored in the password-protected file and data security was ensured.

Statistical analysis

The data were represented as frequency (percentage), mean (standard deviation), median (interquartile range). Linearity of all quantitative data was assessed using Kolmogorov Smirnov analysis and tests of statistical significance (analysis of variance (ANOVA) followed by post hoc Tukey’s Honest Significant Difference (HSD) or Kruskal-Wallis test followed by post hoc Dunnet’s test) were used as per the data type. Receiver operating characteristic (ROC) curve analysis was used to assess the ability of NLR at admission to predict severe COVID-19 or mortality. Ordinal regression analysis was used to assess the impact of individual parameters on disease severity and mortality. SPSS software, version 19.0 (Statistical Package for the Social Sciences Inc, Chicago, IL, USA) was used to carry out statistical analysis. A significance level of <0.05 was considered statistically significant.

## Results

The total study population of 748 subjects was divided into four subgroups depending on the severity of their illness. A maximum number of subjects were found to be having a mild illness (Group B, n=366, 48.93%) while 236 subjects (Group A, 31.55%) were asymptomatic, 75 subjects had moderate symptoms (Group C, 10.02%), and 44 subjects had severe symptoms (Group D, 5.88%). The severity of symptoms was found to be significantly increasing with age (p<0.001), however, four study groups were matched for gender (p=0.074). Symptoms such as fever (p<0.001), dry cough (p<0.001), shortness of breath (p<0.001), and sore throat (p<0.001) were found to be significantly higher in frequency in Groups C and D compared to rest of the groups.

History of hypertension was noted to be significantly higher in Group C and Group D compared to Group A and B (p<0.001). NLR was found to be significantly different between study groups (p<0.001). Further, on post hoc analysis, it was found to be significantly higher in subjects with moderate (Group C, median 3, IQR 3-5) and severe COVID-19 (Group D, median 8, IQR 5-12.5) compared to both asymptomatic (Group A, median 2, IQR 1-2) and mild COVID-19 (Group B, median 2, IQR 1-2). Also, NLR was significantly higher in Group D compared to Group C.

C-reactive protein, lactate dehydrogenase, ferritin, and D-dimer levels were compared between study groups and were found to be significantly different. On post hoc analysis, all three parameters were found to be significantly higher in Group B, C, and D compared to Group A except for ferritin. Also, these levels were significantly higher in Groups C and D compared to Group A. A similar trend of significantly higher levels of these parameters in Group D compared to Group C was observed (Table 1).

**Table 1:** Relationship between demographic and medical parameters with the severity of COVID-19 ^$^One-way ANOVA used, ^#^Chi-square test used, ^^^Kruskal-Wallis test used IHD: ischemic heart disease; CRP: C-reactive protein; LDH: lactate dehydrogenase; NLR: neutrophil-lymphocyte ratio; COVID-19: coronavirus disease 2019

Variables		Total (n=748)	Asymptomatic Group A (n=263)	Mild Group B (n=366)	Moderate Group C (n=75)	Severe Group D (n=44)	P-value
Age (year) Mean (SD)		43.85 (17.60)	38.76 (17.02)	42.96 (16.88)	57.37 (14.17)	60.20 (11.96)	<0.001^$^
Gender							
Male		476 (63.6)	160 (60.8)	229 (62.6)	52 (69.3)	35 (79.5)	0.074
Female		272 (36.4)	103 (39.2)	137 (37.4)	23 (30.7)	9 (20.5)	
Fever		247 (33.0)	5 (1.9)	171 (46.7)	43 (57.3)	28 (63.6)	<0.001^#^
Dry cough		258 (34.5)	4 (1.5)	158 (43.2)	58 (77.3)	38 (86.4)	<0.001^#^
Shortness of breath		133 (17.8)	0 (0.0)	35 (9.6)	55 (73.3)	43 (97.7)	<0.001^#^
Running nose		93 (12.4)	1 (0.4)	68 (18.6)	22 (29.3)	2 (4.5)	<0.001^#^
Sore throat		210 (28.1)	5 (1.9)	157 (42.9)	26 (34.7)	22 (50)	<0.001^#^
Diabetes mellitus		117 (15.6)	24 (9.1)	29 (7.9)	29 (38.7)	35 (79.5)	<0.001^#^
Hypertension		156 (20.9)	29 (11.0)	70 (19.1)	32 (42.7)	25 (56.8)	<0.001^#^
IHD		36 (4.8)	1 (0.4)	12 (3.3)	10 (13.3)	13 (29.5)	<0.001^#^
NLR (Median (IQR))		2 (1-3)	2 (1-2)	2 (1-2)	3 (3-5)	8 (5-12.5)	<0.001^
CRP(mg/dl) (Median (IQR))		4 (1-12)	2 (1-4)	4 (2-9)	49 (36-76)	73 (66.6-91)	<0.001^
LDH (IU/l) (Median (IQR))		177 (162-201)	169 (157-181)	172 (161-188)	321 (287-382)	415 (349-431)	<0.0001^
Ferritin (ng/ml) (Median (IQR))		119 (60-258)	86 (51-196)	90.5 (50.8-173.5)	452.0 (398.0-560.0)	908 (740.8-1010.0)	<0.001^
D-dimer (ng/ml) (Median (IQR))		100 (100-255)	100 (100-100)	172 (161.8-188)	740 (540-920)	1451 (1022-1770)	<0.0001^
Outcome	Discharge	721 (96.4)	263 (100)	366 (100)	75 (100)	17 (38.6)	<0.0001^#^
	Death	27 (3.6)	0	0	0	27 (61.4)	

A strong uphill correlation of NLR was noted with D-dimer levels (r=0.638), however, the moderate uphill correlation was noted with disease severity (r=0.53) and other parameters of disease severity viz. CRP, LDH, and ferritin. All the correlations were found to be statistically significant (p<0.0001) (Table 2).

**Table 2:** Correlation analysis of NRL with disease severity and associated parameters ^^^statistically significant correlation CRP: C-reactive protein; LDH: lactate dehydrogenase; NLR: neutrophil-lymphocyte ratio

		Severity	CRP (mg/L)	LDH (IU/L)	Ferritin (ng/ml)	D-dimer (mcg/ml)
NLR	Correlation Coefficient	0.530^	0.529^	0.586^	0.551^	0.638^
	Sig. (two-tailed) (p-value)	<0.0001	<0.0001	<0.0001	<0.0001	<0.0001

**Figure 2:**
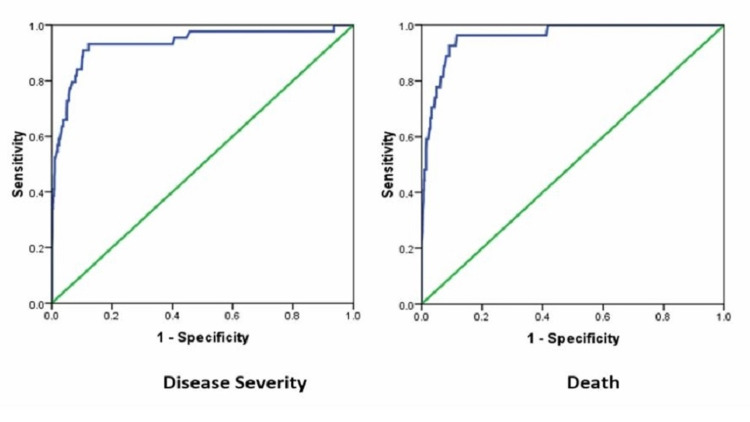
ROC curve analysis to predict NLR as predictor of disease severity (A) and death (B) ROC: receiver-operating characteristic; NLR: neutrophil-lymphocyte ratio

ROC curve analysis revealed NLR to be an excellent predictor of disease severity (sensitivity 93.3% and specificity 87.8 %) at a cut-off of >3.89 as well as a prognostic parameter for risk of death (sensitivity 96.3 % and specificity 88.3%) at cut-off of >4.14 (Table 3, Figures 2A-2B).

**Table 3:** Prognostic significance of NLR at admission to predict disease severity and death in COVID-19 NLR: neutrophil-lymphocyte ratio

Test Result Variable(s)	NLR cut-off	Area	Sensitivity (%)	Specificity (%)	P-value	
						Disease severity	>3.89	0.933	93.2	87.8	<0.0001	
Death	>4.14	0.959	96.3	88.3	<0.0001	

Ordinal logistic regression analysis was performed to predict the risk of contracting severe COVID-19 and mortality depending on various markers at admission. NLR was found to be strongest and only significant independent positive predictor for contracting severe disease (Odd’s ratio 1.396, 95% CI=1.112-1.753, p=0.004) and mortality (Odd’s ratio 1.276, 95% CI=1.085-1.499, p=0.003) (Table 4).

**Table 4:** Ordinal logistic regression analysis to predict the risk of contracting severe COVID-19 and mortality CRP: C-reactive protein; COVID-19: coronavirus disease 2019; LDH: lactate dehydrogenase; NLR: neutrophil-lymphocyte ratio

	Severe COVID-19					Mortality				
Parameter	Odd’s ratio	Low	high	Wald’s X^2^	P-value	Odd’s ratio	Low	high	Wald’s X^2^	P-value
NLR	1.396	1.112	1.753	8.280	0.004	1.276	1.085	1.499	8.719	0.003
CRP	1.039	1.018	1.060	13.590	0.000	1.020	1.003	1.038	5.020	0.025
LDH	0.998	0.992	1.004	0.534	0.465	1.002	0.997	1.007	0.629	0.428
Ferritin	1.000	0.999	1.002	0.241	0.623	1.000	0.999	1.002	0.190	0.663
D-dimer	1.002	1.001	1.004	8.691	0.003	1.002	1.000	1.003	7.346	0.007

## Discussion

NLR is defined as the absolute neutrophil count (ANC)/absolute lymphocyte count (ALC) [16]. Since the beginning of the pandemic, studies have been investigating the role of NLR in COVID-19 prognostication and its utility as a biomarker of severity. NLR has been reported to prognosticate mortality, progression to severe disease, risk of intubation, risk of severe disease in intubated patients, days intubated, ICU admission, and longer intensive care unit (ICU) admission [10-14,17-20]. Two meta-analyses of n=19 and n=13 studies found significant associations between higher NLR and COVID-19 severity and mortality [21,22]. Earlier studies found links to higher NLR in chronic conditions with low-grade inflammatory nature, such as obesity, hypertension, diabetes mellitus, atherosclerotic events of the heart and brain, and various cancers [7-9,23]. NLR might maintain its predictive ability for COVID-19 severity even in patients with comorbidities. For instance, NLR significantly predicted COVID-19 severity and survival in hospitalized patients with different types of cancers [24]. These underlying diseases are risk factors for severe COVID-19. It has been suggested that each increased NLR unit resulted in an 8% higher mortality in COVID-19 patients [25].

Apart from NLR, two other studied markers from complete blood count were platelet to lymphocyte ratio (PLR) and monocyte to lymphocyte ratio (MLR). But out of these, NLR outperformed in prognosticating mortality compared with PLR and LMR [26]. NLR could predict progression to ARDS and the need for mechanical ventilation [27]. There is wide variation in the clinical severity of COVID-19 ranging from an asymptomatic state to severe disease characterized by ARDS, respiratory failure, and MODS. Around 20% of hospitalized cases develop severe disease and in-hospital case fatality due to COVID-19 has been reported as 2-3% by multiple studies [28,29].

Various factors have been proposed to affect disease severity in COVID-19. The present study aimed to assess the association between NLR and severity of disease and mortality in COVID-19. We found a significant difference in NLR levels among discharged and deceased, being significantly higher in later. NLR was significantly higher in severe diseases. There was a significant difference in age, comorbidities, inflammatory markers between discharged and deceased. Our study findings are consistent with the results of previous similar studies. Several meta-analyses have reported that patients with severe COVID-19 infection had a higher NLR than those with non-severe COVID-19 infection [10-14,21,22].

In COVID-19 patients, the absolute value of peripheral white blood cells is usually normal or low, and lymphopenia is common. However, in severe COVID-19 disease, the lymphocyte count decreases progressively, while the neutrophil count gradually increases. This may be due to excessive inflammation and immune suppression caused by the SARS-COV-2 infection [30]. Neutrophils are regarded as proinflammatory cells with a range of antimicrobial activities, which can be triggered by virus-related inflammatory factors, such as IL-6 and IL-8 [31]. On the other hand, systemic inflammation triggered by SARS-COV-2 significantly depresses cellular immunity, leading to a decrease in CD4+ T cells and CD8+ T cells. In addition, SARS-COV-2-infected T cells may also cause cytopathic effects on T cells [32]. The higher NLR resulted from the increased neutrophil count and decreased lymphocyte count. The NLR reflects the balance between innate immunity (neutrophils) and adaptive immunity (lymphocytes). As increased NLR indicates more systemic inflammation, more inflammatory tissue damage, and raised inflammatory markers. In severe cases, the higher NLR indicated that the immune system was dysregulated more severely and could not dampen the overactive innate immune response. These inflammatory overactivation responses might aggravate the production of a cytokine storm and worsen tissue damage [33]. Cytokine storm is the main cause of death in the late stage of SARS-CoV-2 infection [34].

Studies have shown that on-admission NLR could predict COVID-19 prognosis and can be used as a risk stratification tool. This predictive ability increases for a few days after admission when NLR reaches its peak. However, NLR gradually loses its predictive ability as inflammation reduces and the patient recovers from COVID-19 [35,36]. Measurement of NLR requires a complete blood count with differentials, a routine, cheap, widely available, and simple laboratory test. At last, various COVID-19 variants are showing different outcomes of morbidity and mortality [37]. Therefore, we suggest future researchers update the findings related to systemic inflammatory markers specifically for emerging variants.

The main strength of this study was that it tried to highlight a relatively unexplored domain on the prognosis of COVID-19 patients. Further, this research captured COVID-19 patients with a wide range of disease severity and compared NLR levels in patients with different disease severity. Also, many potential predictors of mortality in COVID-19 patients were tested.

Our study has several limitations. Being a single-center study, the study has limited external validity. Further, a single measurement of NLR within 24 hours of hospitalization and a lack of a control group are other limitations. We suggest multicenter studies to clarify the role of NLR in the severity of disease and mortality in COVID-19.

## Conclusions

High NLR was significantly associated with disease severity and mortality in COVID 19 patients in our study. Based on our study, NLR can be used as an early warning signal for severe COVID-19 disease, however further studies are required to validate this finding and it had a significant correlation with other inflammatory markers of COVID-19 severity like CRP, D-dimer, and serum ferritin.

Being a simple, easily available, cost-effective investigation, on-admission NLR could predict COVID-19 prognosis and can be used as a risk stratification tool. In view of various emerging COVID-19 variants with different outcomes of morbidity and mortality, future research is needed to update the findings related to systemic inflammatory markers specifically for emerging variants.
